# Mitochondrial junctions with cellular organelles: Ca^2+^ signalling perspective

**DOI:** 10.1007/s00424-018-2179-z

**Published:** 2018-07-07

**Authors:** Alexei V. Tepikin

**Affiliations:** Department of Cellular and Molecular Physiology, Institute of Translational Medicine, University of Liverpool, Crown Street, Liverpool, L69 3BX UK

**Keywords:** Endoplasmic reticulum, Mitochondria, Organellar junctions, Membrane contact sites, Ca^2+^ signalling, Reactive oxygen species

## Abstract

Cellular organelles form multiple junctional complexes with one another and the emerging research area dealing with such structures and their functions is undergoing explosive growth. A new research journal named “Contact” has been recently established to facilitate the development of this research field. The current consensus is to define an organellar junction by the maximal distance between the participating organelles; and the gap of 30 nm or less is considered appropriate for classifying such structures as junctions or membrane contact sites. Ideally, the organellar junction should have a functional significance, i.e. facilitate transfer of calcium, sterols, phospholipids, iron and possibly other substances between the organelles (Carrasco and Meyer in Annu Rev Biochem 80:973–1000, 2011; Csordas et al. in Trends Cell Biol 28:523–540, 2018; Phillips and Voeltz in Nat Rev Mol Cell Biol 17:69–82, 2016; Prinz in J Cell Biol 205:759–769, 2014). It is also important to note that the junction is not just a result of a random organelle collision but have active and specific formation, stabilisation and disassembly mechanisms. The nature of these mechanisms and their role in physiology/pathophysiology are the main focus of an emerging research field. In this review, we will briefly describe junctional complexes formed by cellular organelles and then focus on the junctional complexes that are formed by mitochondria with other organelles and the role of these complexes in regulating Ca^2+^ signalling.

## Junctions between cellular organelles

The prominent role of junctions between the endoplasmic reticulum (ER) and the plasma membrane (PM) in the regulation of Ca^2+^ signalling and lipid transport has been recently identified (reviewed in [19, 125]). The discovery that a fundamental signalling process—store operated Ca^2+^ entry (SOCE) (reviewed in [69, 99, 128, 132]) requires the direct interaction of two relatively small proteins (STIM and Orai) anchored in different organellar membranes (the ER membrane and the PM membrane [45, 101, 105, 142]) attracted considerable interest from cell physiologists and stimulated interest in the formation of the platforms for such interactions, i.e. ER-PM junctions. SOCE is vital for Ca^2+^ reloading of the ER and for maintaining Ca^2+^ signalling (reviewed in [128]). Other recently identified, specific functions of Ca^2+^ signalling micro-domains generated in the ER-PM junctions (e.g. [81]) further highlighted the importance of these signalling platforms.

cAMP signalling is another signalling modality operating in the ER-PM junctions; studies from the A. Hofer laboratory recently defined a novel mechanism of cAMP signalling SOcAMPS (store-operated cAMP signalling) which is activated by ER Ca^2+^ store depletion and involves the activation of adenylyl cyclase 3 by STIM [97, 107]. Another form of interplay between Ca^2+^ and cAMP signalling in the ER-PM junctions was extensively characterised in a number of elegant papers by D. Willoughby and colleagues from D. Cooper laboratory. This mechanism involves the direct interaction of adenylyl cyclase 8 and Orai1 [169, 170]. In addition to serving as platforms for SOCE and SOcAMPS, the ER-PM junctions play important roles in the transport of phospholipids (e.g. [21, 25, 152]) and sterols [56, 146].

The molecular mechanism of ER-PM junction formation was first discovered and characterised in yeasts where three groups of proteins Ist2, tricalbin proteins Tcb1–3 and Scs2/Sc22 contribute to the tethering of the organelles ([102, 110] reviewed in [135]). Extended synaptotagmins (mammalian analogues of tricalbins) were later shown to mediate the formation of ER-PM junctions in mammalian cells [22, 60]. The ER is a particularly prominent organelle in its ability to form junctions.

ER junctions with endosomes have been described and are important for the regulation of dynamics and fission of these organelles [51, 143, 174]. It is also likely that the ER-endosomal/lysosomal junctional complexes are important for the coordination of Ca^2+^ signalling between these organelles [84, 85, 103, 104, 118]). ER junctions with Golgi are essential for the transfer of lipids between the two organelles [114, 115]. Recent study utilising advanced optical spectral microscopy revealed that ER is the preferred interacting organelle for Golgi, peroxisomes and lipid droplets (LDs) in mammalian cells [159].

Membrane tethering between ER and Golgi is mediated by the oxysterol-binding protein (OSBP), which also serves as a conduit for the transfer of sterols and phospholipids between these two organelles [114, 115].

Direct non-vesicular lipid transfer operates between the ER and peroxisomes [138]. Tethers between these organelles have been visualised in the 80s of the previous century [173]. Recently, the proteins responsible for tethering ER and peroxisomes (Pex3p and Inp1p) have been identified in yeasts [88]. This function in mammalian cells is mediated by ACBD5 and VAPB [28].

Interaction between the ER and LDs is important for the lipid transfer to LDs; a complex consisting of fatty acid transport protein 1 (FATP1) and diacylglycerol O-acyltransferase 2 (DGAT2) have been identified as important for both ER-LD interaction and the lipid loading of LDs [172]. Another protein seipin was recently shown to be important for the ER-LD contacts as well as being involved in lipid and protein delivery from ER to LD [144].

ER contacts with phagosomes generate highly localised Ca^2+^ signals important for phagocytosis [124]. Both junctate and STIM1 are involved in the formation of the junctions between the ER and phagosomes and, interestingly, support different forms of localised Ca^2+^ responses [62, 124].

Junctions between the ER and other cellular organelles are probably the most numerous inter-organellar junctions. However, junctions formed by other organelles have also been described and include contacts of LDs with peroxisomes and lysosomes (reviewed in [54]), and contacts of lysosomes with peroxisomes [24]. Importantly for the purposes of this review many organelles also form contacts with mitochondria.

## Mitochondrial contacts with other cellular organelles

Mitochondria interact and form junctions with LD [6]. Perilipin 5 was shown to be important for this organellar linkage [167]. Another study indicates the importance of mitofusin 2 and perilipin 1 in mediating the interaction between mitochondria and LD [14]. Interestingly, the composition of peridroplet mitochondria and their bioenergetics capacity was shown to be different from their cytoplasmic neighbours [6].

The components of the contact sites between mitochondria and peroxisomes have been characterised using a genome-wide screen in yeast. Pex11 and Mdm34 have been identified as interacting partners involved in the formation of junctions between these cellular organelles [112].

Contacts between mitochondria and Golgi have been described in experimental papers utilising optical microscopy [38, 159]. Interestingly, triple contacts between mitochondria, ER and the Golgi apparatus have been recently identified [159]. Golgi-mitochondrial contacts could be important for Ca^2+^ signalling in both organelles [38].

Mitochondria-lysosome contacts have also been described in mammalian cells [167, 171]. In another study, mitochondria-lysosomal contacts were systematically investigated using a plethora of microscopy and molecular biology techniques. The observed contacts were tight (approximately 10 nm between the membranes of the participating organelles) and were associated with mitochondrial fission [171]. Two proteins, mitochondrial FISI1 and lysosomal RAB7, were reported to regulate the formation and dissolution of the contacts. Specifically, GTP-bound RAB7 induced the formation of contacts, whilst GDP bound RAB7 dissolved contacts. Conversion from GTP bound to GDP bound forms of RAB7 was facilitated by the GTPase-activating protein TBC1D15, recruited to the contact sites by interaction with FISI1 [171]. Notably, the involvement of a RAB GTPase in the formation of the junctions between the vacuole (lysosome-like structure) and mitochondria was earlier demonstrated in yeasts [70].

Direct contact between endosomes and mitochondria is utilised for the iron transfer from transferrin receptor-containing endosomes to the mitochondria [33, 149]. Interestingly, most of the interactions between these two organelles were short-lived (< 0.5 s), illustrating the notion that organellar junctions do not need to be stable or long-lasting to fulfil physiologically important roles [33].

**Fig. 1:**
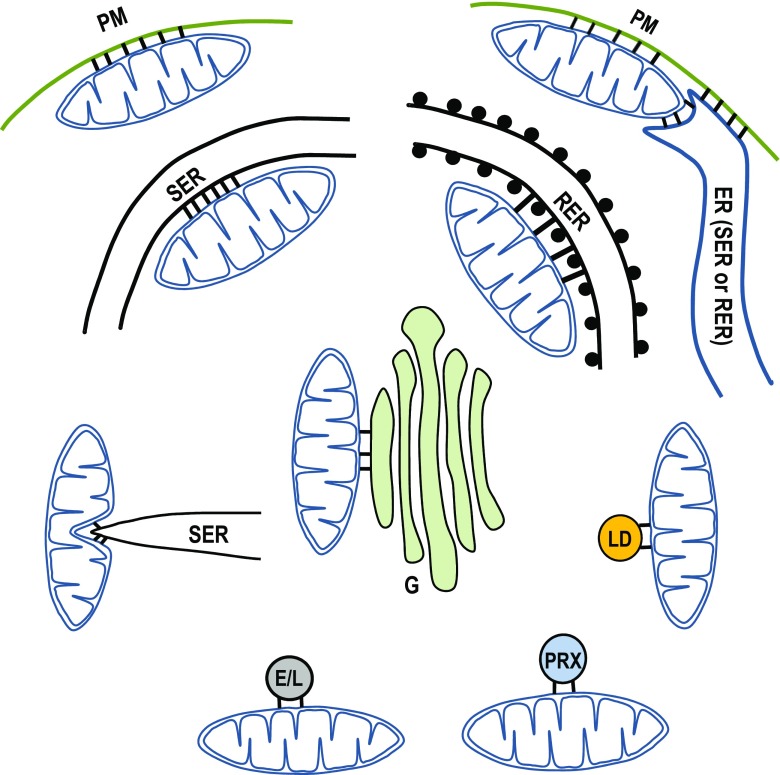
Mitochondrial junctions/interactions with other cellular organelles. Abbreviations in this figure: plasma membrane (PM), endoplasmic reticulum (ER), smooth ER (SER); rough ER (RER); lipid droplet (LD); peroxisome (PRX); endosomes/lysosomes (E/L); Golgi (G). The tethers linking the organelles are indicated by short black bars. Two types of mitochondrial junctions with the SER are included in the figure. The lower mitochondrial-SER junction (SER strand approaches perpendicularly to the mitochondrial outer membrane) illustrates the interaction involved in mitochondrial fission [20, 50, 90]). The upper mitochondrial-SER junction (membranes of the organelles are running parallel to one another) is involved in signalling and lipid transfer between the organelles but not in mitochondrial fission. Note the difference in the length of the tethers between the mitochondrial-SER junctions and mitochondrial-RER junctions (see [29]). A number of triple organellar junctions have been reported (e.g. [159]); in this diagram, we show a putative triple mitochondria-PM-ER junction. Two or three types of tethers could be formed in the triple junctions (two types is the minimal requirement); in this diagram, we show the three types of tethers for illustrative purposes. The strand of ER approaching the PM in the proximity of the ER-PM junction could be SEM [126] or REM [106] but only ribosome-free ER membranes have been shown to form junctions with PM [106, 126]. The properties of the ER and PM in the triple contact regions with mitochondria require further investigations

Mitochondrial junctions with other cellular organelles are schematically illustrated on Fig. 1.

## ER-mitochondria junctions

**Fig. 2:**
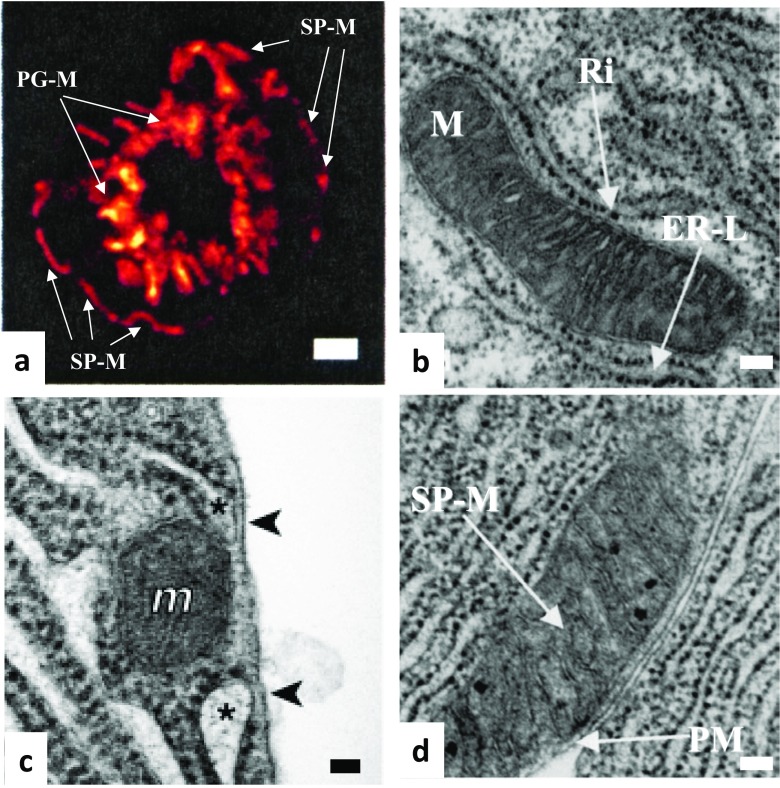
Mitochondria can be found in close proximity to the endoplasmic reticulum and the plasma membrane in pancreatic acinar cells. **a** Images of mitochondria in live pancreatic acinar cells (adapted with modifications from [165]). Mitochondria were loaded with the ΔΨ indicator TMRM (tetramethylrhodamine methyl ester). SP-M indicates subplasmalemmal mitochondria (see also parts **c** and **d** of this figure and [76, 129]). PG-M indicates perigranular mitochondria. In this cell type, PG-M can be found in close proximity to Golgi, ER strands and secretory granules (see [38, 76, 129, 158]). Scale bar corresponds to 4 μm. **b** Example a mitochondrion located in a close proximity to a rough ER strand (adapted with modifications from [76]). Ri indicates ribosome. ER-L indicates the ER lumen. Scale bar represents 100 nm. **c** ER-PM junctions (indicated by arrowheads) with associated mitochondrion (*m*). The image is adapted with modifications from [106]). The lumens of ER strands approaching the plasma membranes are highlighted by asterisks. This image is an example of a triple organellar junction in a primary mammalian cell. Scale bar represents approximately 50 nm. **d** Subplasmalemmal mitochondrion (SP-M) shown with associated plasma membrane (PM) region. Note the strands of the rough ER in close proximity to the mitochondrion on the other side from the PM. Scale bar corresponds to approximately 100 nm. The figure was adapted with modifications from [76]

Early indications of connections between these two organelles have been published in the 1950s of the previous century [9, 26]. Considering the short distance (< 30 nm) between cellular organelles that should be bridged by tethers to form the junctions, electron microscopy (EM) technique is the preeminent methodology in this rapidly developing research field. The contacts between mitochondria and the ER have been indeed visualised by EM (an example is shown on the Fig. 2); furthermore, tethers between the two organelles were also documented in experiments utilising electron tomography [29]. The length of the tethers between strands of smooth ER and mitochondria was approximately 10 nm, whilst the distance bridged by the tethers connecting rough ER and mitochondria was approximately 25 nm [29]. This and other EM studies complemented biochemical observations that a specific fraction of the ER is associated with mitochondria. This fraction is termed MAM (mitochondria-associated membranes). It is important for phospholipid synthesis and the transport of phospholipids between the ER and mitochondria, including the transfer of phosphatidylserine from the ER to mitochondria and of phosphatidylethanolamine from mitochondria to the ER (early evidence [161], recent reviews [98, 132, 162]). The specific biochemical procedures involved in the isolation of MAMs are described in a recent review by J. Vance [162]. Importantly, in most cell types, MAMs are identified/characterised by proteins that are not unique in MAMs but are enriched in MAMs [162]. These proteins include phosphatidylserine synthase-1 and synthase-2 [153], Sigma-1 receptor [65], Mitofusin 2 [34] and, importantly for this review, inositol trisphosphate receptors (IP3R) [140, 154].

Recently, there was considerable progress in the characterisation of the molecular composition of the tethers linking ER with the outer mitochondrial membrane (OMM). In yeasts, a complex termed ERMES (ER-mitochondria encounter structure) has been identified by exceptionally elegant experiments combining expression of artificial tether linking ER with mitochondria and analysis of mutations in yeasts colonies. These experiments identified components of ERMES on the basis that mutations or deletions of these components results in growth deficiency that could be rescued by the expression of the artificial tether [89]. The identified in these experiments proteins Mmm1/Mdm10/Mdm12/Mdm34 form ERMES. Two proteins forming ERMES (Mdm34 and Mdm10) are anchored in OMM; whereas Mmm1 is an ER membrane resident and Mdm12 is a cytosolic protein recruited to ERMES complex ([89] reviewed in [95]). A recent study by S. Kawano and colleagues reported that the Mmm1-Mdm12 complex is sufficient for the transfer of phospholipids between membranes [82]. Notably, in yeasts, there is a redundancy of both lipid ER-mitochondria tethering and lipid transfer mechanisms; in particular, conserved EMC (endoplasmic reticulum membrane protein complex) has been identified and suggested to mediate both tethering and lipid transfer functions [94] (reviewed in [119]). Another redundancy is based on the functional substitution of ER-mitochondrial junctions in ERMES impaired yeasts with junctions formed between vacuole and mitochondria termed vCLAMP (vacuole-mitochondria contact patches) [44, 70]). Impairment of ERMES results in the expansion of vCLAMP and vice versa, whereas the elimination of both structures is lethal. Vps39 was shown to be important for vCLAMP formation [44, 70]. Recent studies from the B. Kornmann laboratory indicated that endosomal protein Vps13 and mitochondrial protein Mcp1 mediate the functions of vCLAMP [75, 96].

ERMES complex/components are not retained in metazoans and other types of proteins are responsible for the formation of ER-mitochondria junctions in animal cells. Linkers formed by OMM protein VDAC1 (voltage-dependent anion channel 1), IP3R (located in the ER) and a molecular chaperone glucose-regulated protein 75 (grp75) was suggested by G. Szabadkai and colleagues from R. Rizzutto laboratory [154]. The presence of IP3Rs was later observed in proximity-labelling assays designed to reveal the proteome of ER-mitochondrial junctions [23]. The composition of such linker is clearly beneficial for the Ca^2+^ transfer from the ER to mitochondria. Mitofusin 2 was suggested as the linker between the ER and mitochondria [34]. This notion has been challenged (e.g. [27, 46]) and the debate currently continues [47, 48, 121, 122]. Recently, a number of ER proteins interacting with mitochondrial proteins and therefore capable, in principle, to serve as tethers have been reported; they include: vesicle-associated membrane protein-associated protein B (VAPB) interacting with mitochondrial protein tyrosine phosphatase interacting protein 51 (PTPIP51) [36]; oxysterol-binding proteins (OSBP)-related proteins ORP5 and ORP8 that also interact with PTPIP51 [52]; Bap31 that interact with mitochondria-located Fission 1 [174] and ribosome-binding protein 1 (RRBP1) with its mitochondrial binding partner Synaptojanin-2-binding protein (SYNJ2BP) [73].

Among the regulators of ER-mitochondrial junctions are ER-shaping proteins reticulons, which were identified as a result of ascorbate peroxidase proximity labelling [23]. The ability of reticulons (specifically of RTN1A, RTN2B, and RTN3B) to increase ER-mitochondria interaction was determined by split luciferase assay [23]. Similar technique was used to ascertain the role of receptor expression-enhancing protein 1 (REEP1) in potentiating ER-mitochondria interaction [100]. Another regulator of ER-mitochondrial junctions is the endoplasmic-reticulum-associated E3 ubiquitin ligase Gp78, which is particularly important for rough ER-mitochondria contacts [168].

Mitochondrial fission requires the formation of specific contacts between mitochondrial membrane and the ER. Dynamin related protein 1 (Drp1) is essential for mitochondrial fission (e.g. [92, 151] reviewed in [160]). A study by J. Friedman and colleagues from the G. Voeltz laboratory reported that the location of mitochondrial division is determined by contact with the ER tubule, which is formed before the recruitment of Drp1 to the inter-organellar contact and induces mitochondria constriction at the contact region [50]. The ER in the junction is enriched with inverted formin 2 (INF2) which induces actin filament formation in the junction [90]. Notably, INF2-induced actin recruitment is important for the formation of contacts between the ER and mitochondria [20]

## ER-mitochondria junctions as signalling nanodomains

Ca^2+^ signalling is an important signalling modality operating in the ER-mitochondria junctions. Many biophysical properties of mitochondrial Ca^2+^ influx and extrusion have been characterised in the second part of the twentieth century. In particular it was established that the mitochondrial Ca^2+^ influx system operates as “uniporter” (i.e. does not involve accompanying transfer of other ions for charge compensation) and that it can be efficiently inhibited by Ruthenium Red (RuRed). The mitochondrial Ca^2+^ export system was characterised as Na^+^/Ca^2+^ exchanger, which can also transport Ca^2+^ when Na + is substituted by Li + and can be inhibited by CGP-37157. These crucially important early discoveries are reviewed in [18]. Development of mitochondria specific bioluminescent probes by R. Rizzuto, T. Pozzan and their colleagues has given considerable impetus to the advancement of this research field [139]. Important studies defining the physiological and pathophysiological role of mitochondrial Ca^2+^ have also been conducted in the last three decades of the twentieth century. R. Denton’s group defined an important role of mitochondrial Ca^2+^ in the regulation of the Krebs cycle (reviewed in [37, 113]). At the cellular level, changes in the activity of the Krebs cycle can be visualised by recording NAD(P)H and FAD fluorescence (reviewed in [42]). Clear correlation between cytosolic Ca^2+^, mitochondrial Ca^2+^ and NADH responses has been indeed recorded (e.g. [63, 164]. The upregulation of Krebs cycle and other Ca^2+^-dependent mitochondrial reactions underpins the regulation of mitochondrial ATP production required for efficient stimulus-metabolism coupling (e.g. [77, 157, 166], reviewed in [55, 156]).

The role of Ca^2+^ microdomains and the importance of the contacts between the ER and mitochondria for mitochondrial Ca^2+^ influx was emphasised by studies from the T. Pozzan laboratory [140, 141]. The importance of the microdomains and organellar contacts was attributed to the relatively low affinity of the mitochondrial uniporter to the cytosolic Ca^2+^ [140, 141]. The recent rapid development of this research area provided mechanistic explanation to this phenomenon. The direct electrophysiological recordings of the MCU current were reported by Y. Kirichock and colleagues from the D. Clapham laboratory [86]. In 2011, two laboratories independently identified the protein mediating mitochondrial Ca^2+^ entry and termed it mitochondrial calcium uniporter (MCU) [4, 35]. Approximately 1 year earlier, F. Perocchi and colleagues from the V. Mootha laboratory discovered an important regulator of mitochondrial Ca^2+^ import, MICU1 [131]. This research area undergone rapid development in the next few years and a number of other regulators of MCU have been discovered including MICU2 [134] and EMRE [145]. Both MICU1 and MCU2 are EF hand-containing Ca^2+^-binding proteins [131, 134]. An important role of MICU1 and MICU2 in the MCU complex is to form and regulate the threshold of cytosolic Ca^2+^, which allows efficient Ca^2+^ entry into the mitochondria (e.g. [31, 78, 80, 109, 130] for review see [79] and the paper by C. Mammucari and colleagues in the current issue). A resting mitochondrial membrane potential (ΔΨ) of approximately − 160 mV is sufficient to drive Ca^2+^ entry into the mitochondria even at low resting cytosolic Ca^2+^ concentrations. Increased Ca^2+^ threshold for the mitochondrial Ca^2+^ entry is beneficial for the cell since it prevents or reduces Ca^2+^ entry into the mitochondria at low (resting or near-resting) cytosolic Ca^2+^ levels. This prevents futile Ca^2+^ cycle and the associated bioenergetics costs required to maintain acceptably low mitochondrial Ca^2+^ concentration. Such futile Ca^2+^ cycle and ATP expenditure were recently demonstrated in cells harbouring *MICU1* mutation by G. Bhosale and colleagues from M. Duchen’s laboratory [10]. Threshold created by MICU1 and MICU2 is an important mechanism for reducing the signal-to-noise ratio for the communication between Ca^2+^ signalling and mitochondria. Importantly, it works in conjunction with Ca^2+^ signalling microdomains formed in the ER-mitochondrial junctions, which further increase the difference between bulk cytosolic Ca^2+^ rise and the Ca^2+^ rise in the proximity to the Ca^2+^-releasing channels and OMM region located in the junctional complex. Direct measurements of Ca^2+^ increases in the ER-Mitochondrial junctions have been conducted by G. Csordas and colleagues from the G. Hajnoczky laboratory by placing Ca^2+^ indicators into the junctions [30]. This study reported high amplitude IP3-induced Ca^2+^ responses (> 9 μM) in the junctions (substantially higher than the bulk cytosolic Ca2+ increase) and the relative insensitivity of the junctional Ca^2+^ transients to slow Ca^2+^ buffering by EGTA [30]. The substantial difference between local Ca^2+^ signals in the junction and the rest of the cytosol enhances the signal-to-noise ratio for mitochondrial transfer of Ca^2+^ signals and facilitates this form of stimulus—metabolism coupling. The findings reported by G. Csordas and colleagues were consistent with results reported by M. Giacomello and colleagues who targeted Ca^2+^ indicator to the OMM and reported the appearance of Ca^2+^ hot spots where the Ca^2+^ concentration was found to be more than 5 times higher than that of the bulk cytosolic concentration [57]. The presence of IP3Rs in MAMs and their suggested role as a component of the junctional complex [154] are also in agreement with these findings.

RyRs form another group of intracellular Ca^2+^-releasing channels particularly prominent in the sarcoplasmic reticulum (a specialised form of the endoplasmic reticulum present in muscle cells). There is now a sufficient body of evidence supporting the formation of SR-mitochondrial junctions and privileged local Ca^2+^ transfer from RyR into the mitochondria. Electron microscopy imaging revealed close contacts between mitochondrial and SR membranes (e.g. [66]). High Ca^2+^ concentration hot-spots (> 20 μM) have been recorded on the OMM of cardiomyocytes [39]. Mitochondrial Ca^2+^ increase following RyRs activation occurs in the presence of cytosolic calcium buffer in cardiac [148, 155] and skeletal [150] muscle cells, confirming the existence of functionally coupled organellar junctions. The Ca^2+^ transfer by this mechanism is therefore important for stimulus-metabolism coupling in muscle cells ([16, 155] reviewed in [43]).

Mitochondrial Ca^2+^ transfer in the junctional complexes is important not only for the stimulus-metabolism coupling. A recent study by R. Chakrabarti and colleagues highlighted the importance of Ca^2+^ influx in ER-mitochondrial junction and Ca^2+^ entry into the mitochondria via MCU for mitochondrial fission [20].

Mitochondrial Ca^2+^ is important for the opening of the mitochondrial permeability transition pore (MPTP). MPTP is a high conductance mitochondrial channel permeable to molecules with molecular weight up to 1.5 kDa [40]. The exact role of mitochondrial Ca^2+^ as permissive or initiating factor in physiological/pathophysiological settings involving MPTP is debated (see [8]). Permissive or inducing, the mitochondrial Ca^2+^ is important for MPTP opening and therefore for the associated cell/tissue damage. Considering the importance of MPTP in pathophysiology of cardiovascular system (reviewed in [64]) and nervous system (reviewed in [41]), and the significance of ER-Mitochondrial junctional complexes for mitochondrial Ca^2+^ transfer, one can expect that the role of junctional complexes in pathophysiological conditions will gain considerable attention in the next few years. This process has already began: e.g. a study by L. Hedskog and colleagues suggested the link between the increase in the number of the ER-mitochondrial contacts and the pathophysiology of Alzheimer disease [67], whilst X. Qiao and colleagues highlighted the importance of PTPIP51 (protein regulating ER-mitochondria junction) for ischemia/reperfusion injury [136]. It is safe to predict that the study of the structure, dynamics and role of junctional complexes in diseases will be an important subfield in modern biomedical research.

ER-mitochondrial junctions are also sites of localised H_2_O_2_ nanodomains that were recently directly measured and reported by D. Booth and colleagues [12]. In this elegant study from the G. Hajnoczky laboratory, the authors targeted the H_2_O_2_ sensor HyPer [5] to the inducible linkers between the ER and mitochondria, and observed Ca^2+^-dependent redox nanodomains in the junctions between the organelles [12]. Interestingly, H_2_O_2_ transients potentiated ER Ca^2+^ release [12]. Redox regulation of IP3Rs is well documented (e.g. [2, 13] reviewed in [1]) and junctional complexes involving the ER with a ROS producing organelle (i.e. mitochondrion) is prime location for such regulation. Importantly, RyR are also redox sensitive channels (reviewed in [68]) and important sensitivity adjustment of this channel could take place in SR-mitochondrial junctions by locally produced ROS.

## Mitochondria-PM junctions and Ca^2+^ signalling

The mechanisms tethering mitochondria to the plasma membrane have been characterised in yeasts, where the Num1/Mdm36 anchors ER-mitochondria complex to the plasma membrane [87, 93, 133]. Subplasmalemmal mitochondrial groups have been reported in a number of mammalian cell types (e.g. [49, 76, 129, 137] see also Fig. 2) but the mechanism involved in the formation of tethers between the mitochondria and the plasma membrane in mammalian cells is currently unknown.

Using Ca^2+^ indicators targeted to OMM and the cytosol, Giacomello and colleagues established that mitochondria adjacent to the plasma membrane did not show preferential Ca^2+^ uptake upon activation of store operated Ca^2+^ entry [57]. Furthermore, Ca^2+^ entry via SOCE was ineffective in producing Ca^2+^ hot spots on the OMM. Nevertheless, Ca^2+^ entry into the mitochondria was recorded in these experiments and the peak mitochondrial Ca^2+^ concentration was approximately one order of magnitude higher than in the cytosol [57]. The absence of privilege communication between STIM–Orai channels and mitochondria was also observed in COS-7 cells by M. Korzeniowski and colleagues from the A. Spat laboratory [91].

On the other hand, a study by P. Varadi and colleagues demonstrated that the re-localisation of mitochondria from the plasma membrane results in a clearly resolvable decrease of store operated Ca^2+^ entry and reduction in mitochondrial Ca^2+^ responses [163]. This is consistent with the findings by A. Quintana and colleagues from the M. Hoth laboratory which revealed the prominent role of mitochondria in the immunological synapse. In this highly specialised signalling region, essential for T cell activation, mitochondria regulate store operated Ca^2+^ entry [137]. Importantly, this is achieved by a specialised group of subplasmalemmal mitochondria. The authors concluded that the local subplasmalemmal mitochondria prevent calcium-dependent inactivation of ORAI channels in the immunological synapse and therefore extend/amplify Ca^2+^ responses. This is achieved as a result of a local coordination of STIM/Orai channels, mitochondria and Ca^2+^ pumps of the plasma membrane. This study extends previous findings of the importance of mitochondria in the regulation of SOCE and its electrophysiological manifestation Ca^2+^ release-activated Ca^2+^ (CRAC) current (ICRAC) ([3, 53, 58, 59, 61, 71, 72, 108, 111] and specifically about the role of mitochondrial Ca^2+^ buffering in the regulation of ICRAC inactivation [127]). It is conceivable that mitochondria could regulate SOCE/ICRAC not only via local Ca^2+^ uptake but also by releasing products of mitochondrial metabolism in the proximity to the Ca^2+^ channel. In particular, it was shown that ATP released from subplasmalemmal mitochondria can facilitate SOCE by providing local Ca^2+^ buffering [116]. Notably, subplasmalemmal ATP microdomains have been recorded [83]. The authors of this study also suggested that a specific peripheral group of mitochondria is responsible for such micro domains.

Interestingly, the T.Pozzan group demonstrated privilege communication between mitochondria and voltage-gated Ca^2+^ channels. This study reported that the subplasmalemmal mitochondria are exposed to higher Ca^2+^ concentrations and show stronger Ca^2+^ responses than mitochondria located in the deeper regions of the cytoplasm [57]. Similar conclusion was reached in the study by Montero and colleagues that demonstrated very strong Ca^2+^ increases (hundreds of μM) in a subgroup of mitochondria upon activation of voltage-gated Ca^2+^ channels [117]. Interestingly, this study suggests a triple functional interaction between voltage-gated Ca^2+^ channels, RyR and mitochondria [117]. A recent study by A. Valm and colleagues, utilising high-resolution optical microscopy, identified a number of close contacts formed by ERMCSs (ER mitochondria contact sites) with other organelles [159]. The structure-function relationships of such triple organellar junctions will probably form an exciting avenue for further development in this research subfield.

Subplasmalemmal mitochondria regulate not only Ca^2+^ channels but also Ca^2+^ pumps. This coordinated regulation is probably needed to ensure balance between Ca^2+^ signalling and Ca^2+^ homeostasis. The important role of subplasmalemmal mitochondrial group in the regulation of both SOCE- and PMCA-mediated Ca^2+^ fluxes was reported M. Frieden and colleagues [49].

Mitochondria are an important source of reactive oxygen species (reviewed in [15, 120, 147]). Both Ca^2+^ extrusion by PMCA and Ca^2+^ entry via STIM/Orai channels are redox sensitive processes (e.g. ([11, 17], see also [123]). Recently, mitochondrial ROS was implicated in the regulation of SOCE [7]). Subplasmalemmal mitochondria would be particularly suitable organelles for this form of regulation.

## Concluding remarks

One can observe clear indications of the emergence of a new research field focused on the mechanisms contributing to the formation of junctions between cellular organelles and determining functions of the interorganellar complexes. The development of this field is facilitated by the rapid advances in super-resolution microscopy and correlative optical-electron microscopy. This emerging field has already facilitated the development of new molecular biology techniques (e.g. introduction of artificial tethers/linkers that can bridge cellular organelles and can be decorated with sensors of signalling molecules). Development of techniques for selective labelling of junctional proteins and consequently identification of junctional proteomes should provide further impetus to this research area. It is likely that ER-mitochondrial junctions and mitochondria-PM junctions serve as important elements in stimulus-metabolism coupling and that this and other physiological functions of the junctional complexes will be actively investigated in the near future. Understanding the mechanisms involved in the formation and functioning of junctional complexes (and particularly of mitochondrial junctions with other cellular organelles) will be beneficial for elucidating the pathophysiological implications of the disruption of these important transport/signalling platforms.
